# Editorial: Frontiers in *Wolbachia* biology 2023

**DOI:** 10.3389/fmicb.2024.1513314

**Published:** 2024-11-11

**Authors:** Takema Fukatsu, Yuval Gottlieb, George Tsiamis, Elizabeth McGraw, Steve Perlman, Didier Bouchon, Karyn Johnson, Mark J. Taylor

**Affiliations:** ^1^Bioproduction Research Institute, National Institute of Advanced Industrial Science and Technology, Tsukuba, Japan; ^2^Graduate School of Life and Environmental Sciences, University of Tsukuba, Tsukuba, Japan; ^3^Department of Biological Sciences, Graduate School of Science, The University of Tokyo, Tokyo, Japan; ^4^Koert School of Veterinary Medicine, Robert H. Smith Faculty of Agriculture, Food and Environment, The Hebrew University of Jerusalem, Rehovot, Israel; ^5^Department of Sustainable Agriculture, University of Patras, Agrinio, Greece; ^6^Department of Biology, The Pennsylvania State University, University Park, PA, United States; ^7^Department of Biology, University of Victoria, Victoria, BC, Canada; ^8^Ecologie et Biologie des Interactions, Université de Poitiers, Poitiers, France; ^9^Australia School of Biological Sciences, The University of Queensland, Brisbane, QLD, Australia; ^10^Department of Tropical Disease Biology, Liverpool School of Tropical Medicine, Liverpool, United Kingdom

**Keywords:** *Wolbachia*, symbiont, genome, insect, nematode

*Wolbachia* is likely the most successful endosymbiotic bacteria associated with insects and other arthropods, as well as nematodes (Werren et al., 2008). Over the past several decades, its widespread presence across the vast range of arthropod in the terrestrial ecosystem, as well as its various biological attributes, have led to an explosive development in *Wolbachia* research. These include the induction of striking reproductive phenotypes, namely cytoplasmic incompatibility (CI), male-killing (MK), feminization (Fem) and parthenogenesis induction (PI) (Kaur et al., 2021); obligatory and conditional beneficial fitness consequences such as nutrient provisioning and resistance to parasites, pathogens and viruses (Hamilton and Perlman, 2013; Pimental et al., 2021); essentiality for host growth, development and survival (Zug and Hammerstein, 2015; Taylor et al., 2005); and others. The biology of *Wolbachia* is not only a captivating area of basic research that covers host-microbe interactions ranging from cell-biology and physiology to ecology and evolution (Serbus et al., 2008; Sanaei et al., 2021), but it is also an important applied research field that contributes to *Wolbachia*-mediated control of vector-borne infectious diseases and pests (Iturbe-Ormaetxe et al., 2011; Ross et al., 2019), and *Wolbachia*-targeted prevention and remedy of filariasis (Taylor et al., 2005; Johnson et al., 2021).

Since the 1st *Wolbachia* Conference held at Crete, Greece, in 2000, *Wolbachia* Conferences have biennially provided a very active and important forum for world's researchers working on *Wolbachia* and other microbial symbionts of arthropods, nematodes etc. The 11th *Wolbachia* Conference was initially planned to be held on 5th-10th July 2020, but because of the COVID pandemic, it was finally postponed to 11th-16th June 2023 and held so successfully. In collaboration with the 11th *Wolbachia* Conference, the Research Topic “*Frontiers in Wolbachia Biology 2023*” was launched to provide a forum to overview achievements recently emerging in this research field. In total, 12 original research articles and two review articles are compiled in the Research Topic, which cover a variety of topics regarding the *Wolbachia* biology.

The reproductive manipulations induced by *Wolbachia* infection are among the focal Research Topics of the *Wolbachia* biology. While several (putative) effector proteins responsible for CI, MK and PI phenotypes have been uncovered for several insect systems including fruit flies, mosquitoes, moths and wasps (Beckmann et al., 2017; LePage et al., 2017; Perlmutter et al., 2019; Katsuma et al., 2022; Arai et al., 2023; Fricke and Lindsay, 2024), considering the diversity of the effector molecules (Cifs, Oscar, Wmk, Pifs, etc.), more extensive survey is needed for understanding the diversity and commonality of the *Wolbachia*-induced reproductive phenotypes. Pramono et al. reported a CI-inducing *Wolbachia* genome from a leaf-mining pest fly *Liriomyza trifolii*. Grève et al. reported the genomes of three feminizing *Wolbachia* strains from the pill bug *Armadillidium vulgare*. Beckmann et al. attempted a modeling approach using an evolutionary algorithm as to how CI-inducing and rescuing proteins evolve. These studies provide basic information toward our better understanding of the biology of *Wolbachia* and its intricate reproductive phenotypes.

*Wolbachia*-mediated control measures against mosquito-borne diseases comprise the recent focal topic in this research field, which are mainly based on *Wolbachia*-mediated suppression of pathogen infections and CI-driven spread of *Wolbachia* infection into host populations (Iturbe-Ormaetxe et al., 2011; Ross et al., 2019). Tafesh-Edwards et al. investigated innate immune responses of the fruit fly *Drosophila melanogaster* after Zika virus infection. They found that some immune-related genes such as *drosocin* and *puckered* are upregulated in a female specific manner, whereas the activity of RNA interference and Toll signaling remain unaffected. Minwuyelet et al. reviewed previous studies on utilization of *Wolbachia* for reducing the transmission of mosquito-borne diseases. Guo et al. also overviewed the *Wolbachia*-mediated technologies for suppressing the transmission of mosquito-borne pathogens, and argued application of the technologies to control of rice pest planthoppers.

*Wolbachia* is difficult to culture *ex vivo*. Thus far, several *Wolbachia* culture systems have been developed, where the fastidious endosymbiotic bacteria can be maintained only when co-cultured with insect cell lines (Masson and Lemaitre, 2020). On the other hand, it was reported that *Wolbachia* purified from insect cells could be maintained in cell-free culture media for at least 1 week without loss of viability or infectivity (Rasgon et al., 2006). Here Behrmann et al. monitored the proliferation of a *Wolbachia* isolate from the mosquito *Aedes albopictus* in a host cell-free *in vitro* culture system by quantitative PCR. By supplementing a mosquito cell membrane fraction and fetal bovine serum, extracellular *Wolbachia* replication for up to 12 days was detected. Notably, even after the *ex vivo* maintenance for 12 days, the *Wolbachia* cells could establish infection to a fresh mosquito cell line, suggesting the possibility that *Wolbachia* might be amenable to some experimental or genetic manipulations using such cell-free culture systems.

Population dynamics of *Wolbachia* within host cells and tissues is important for understanding phenotypic consequences of the symbiont infection such as fitness effects, intensity of reproductive manipulation, level of reproductive performance, and others (López-Madrigal and Duarte, 2019). Sharmin et al. attempted to elucidate the molecular and cellular mechanisms involved in regulation of intra-host *Wolbachia* titer by adopting chemical and genetic approaches using *Drosophila* fruit flies. In total, 37 chemical inhibitors targeting 14 host cellular/molecular processes, which were reported to affect intracellular bacterial abundance in previous studies, were administrated to *D. melanogaster* and *D. simulans*, and examined for their effects on *Wolbachia* titers. Finally, 5 compounds were identified to significantly increase the intra-host *Wolbachia* titers, which were associated with host Imd signaling, Calcium signaling, Ras/mTOR signaling, and Wnt signaling functions, suggesting that these host mechanisms may negatively regulate the *Wolbachia* titers. By making use of ample molecular genetic tools available for *D. melanogaster*, genetic disruption assays confirmed that disruption of Wnt and mTOR pathways upregulates the *Wolbachia* titers, uncovering that interactions of Wnt and mTOR pathways with autophagy may underlie the negative regulation over *Wolbachia* population. Poulain et al. reported detailed population dynamics of the *Wolbachia* strain *w*Cle associated with the common bedbug *Cimex lecturalius*. In the bedbug, the *Wolbachia* cells densely and endocellularly populate the well-developed symbiotic organs, called the bacteriomes, where the specific *Wolbachia* strain synthesizes B vitamins that are deficient in host's blood meal (Hosokawa et al., 2010; Nikoh et al., 2014). In this context, the population dynamics data of *w*Cle in the bedbug will provide insight into how the exceptional *Wolbachia* strain that turned into an obligatory nutritional mutualist contributes to survival and proliferation in the life cycle of the blood-sucking insect pest that is recently re-emerging worldwide (Doggett and Lee, 2023). Giordano et al. investigated reproductive performance of the soybean aphid *Aphis glycines* infected with or without the facultative symbionts *Arsenophonus* and/or *Wolbachia* under different aphid and soybean genotypes. These studies highlight how *Wolbachia* population is controlled and integrated into the endosymbiotic system in cellular, physiological and ecological contexts.

Since *Wolbachia* is indispensable for survival and reproduction of filarial nematodes, *Wolbachia*-targeting drugs are regarded as promising for medical prevention and remedy of filariasis (Johnson et al., 2021). Hegde et al. reported that an azaquinazoline anti-*Wolbachia* agent, AWZ1066S, facilitates the sterilizing and curing effects of benzimidazole in an experimental model of filariasis. Wangwiwatsin et al. reported *in silico* screening of co-evolving protein sequences between host filarial nematodes and their *Wolbachia* endosymbionts, which identified candidate “hub” genes that may connect multiple host-symbiont interactions and thus may provide potential drug targets via disruption of host-*Wolbachia* interactions.

For genomic and transcriptomic studies, the preparation of *Wolbachia*-derived DNA/RNA usually suffers heavy contamination of host-derived DNA/RNA due to the endosymbiotic nature of *Wolbachia*. Cantin, Dunning Hotopp et al. reported an improved procedure for metagenomic assembly of *Wolbachia* genome through selective enrichment of bacterial DNA from nematode host DNA using ATAC-seq technique. Cantin, Gregory et al. also reported an improved procedure for dual RNA-seq in filarial nematodes and *Wolbachia* endosymbionts using RNase H based ribosomal RNA depletion. These technical improvements may be useful for *Wolbachia* studies of not only nematodes but also insects and other arthropods.

In conclusion, the Research Topic provides a valuable overview of the recent research progress in the field of *Wolbachia* biology. We hope that this Research Topic shows future directions of this research field, which will be manifested in the forthcoming 12th *Wolbachia* Conference to be held at Okinawa, Japan, from 13th to 19th April 2025 (https://web.tuat.ac.jp/~insect/wolbachia2025/).
